# Micro- and Nanoscale Spectroscopic Investigations of Threonine Influence on the Corrosion Process of the Modified Fe Surface by Cu Nanoparticles

**DOI:** 10.3390/ma13204482

**Published:** 2020-10-10

**Authors:** Dominika Święch, Czesława Paluszkiewicz, Natalia Piergies, Ewa Pięta, Kamila Kollbek, Wojciech M. Kwiatek

**Affiliations:** 1 Faculty of Foundry Engineering, AGH University of Science and Technology, al. Mickiewicza 30, 30-059 Krakow, Poland; 2 Institute of Nuclear Physics Polish Academy of Sciences, PL-31342 Krakow, Poland; Czeslawa.Paluszkiewicz@ifj.edu.pl (C.P.); natalia.piergies@ifj.edu.pl (N.P.); ewa.pieta@ifj.edu.pl (E.P.); Wojciech.Kwiatek@ifj.edu.pl (W.M.K.); 3 Academic Centre for Materials and Nanotechnology, AGH University of Science and Technology, al. Mickiewicza 30, 30-059 Krakow, Poland; kamila.kollbek@agh.edu.pl

**Keywords:** surface-enhanced infrared absorption spectroscopy (SEIRA), surface-enhanced Raman spectroscopy (SERS), copper nanoparticles (NPs), threonine (Thr), corrosion

## Abstract

The work presents a comprehensive vibrational analysis of the process of adsorption of threonine (Thr) onto an Fe surface with deposited Cu nanoparticles (NPs) (of about 4–5 nm in size) in a corrosive environment. The application of surface-enhanced Raman spectroscopy (SERS) and surface-enhanced infrared absorption spectroscopy (SEIRA) provides the opportunity for detailed description of adsorption geometry of amino acid onto a metal surface. The combination of conventional infrared spectroscopy (IR) with atomic force microscopy (AFM) resulted in a nano-SEIRA technique which made it possible to provide a precise description of adsorbate binding to the metal surface. The studies presented confirmed that there is a very good correlation between the spectra recorded by the SERS, SEIRA, and nano-SEIRA techniques. Threonine significantly influenced the process of corrosion of the investigated surface due to the existing strong interaction between the protonated amine and carboxylate groups and the CuNPs deposited onto the Fe surface. In addition, the application of two polarization modulations (*s* and *p*) in nano-SEIRA allows subtle changes to be observed in the molecule geometry upon adsorption, with the carboxylate group of Thr being almost horizontally oriented onto the metal surface; whereas the amine group that contains nitrogen is oriented perpendicular to this surface.

## 1. Introduction

Metal materials are exposed to aggressive media and are prone to corrosion. A lot of corrosion inhibitors used for the inhibition of corrosion cause health hazards [1,2]. The use of organic inhibitors, such as amino acids, has many advantages. Amino acids are biogenic, nontoxic, biodegradable, and have been used in various applications for many years [2,3,4,5,6,7,8,9]. Providing a description of the process of adsorption of amino acids onto metallic surfaces in an aggressive environment is of great significance in order to understand the mechanism of their anticorrosive effect [4,5,6]. There is a lot of research in which amino acids have been reported to be an effective corrosion inhibitor in various aggressive media [3,7,10,11]. For example, the corrosion inhibition effect of threonine (Thr) on the corrosion of copper (Cu) in hydrochloric acid (HCl) has been investigated by different research groups [10,11]. The results obtained by Zhang et al. indicated that 10^−3^ M Thr has good inhibition efficiency (I_E_) for copper in 0.5 M HCl solution (I_E_ = 83.4) [10].

In recent years, there has been a growing interest in the use of metallic nanoparticles of less than 100 nm in size [12,13,14,15]. Nanoparticles (NPs) have chemical and physical characteristic properties ascribed to their comparatively small size and high surface-area-to-volume ratio. Copper nanoparticles—amongst various metallic NPs—show, i.e., high selectivity [16], catalytic activity [17,18], very good electrical conductivity [19], as well as antibacterial capacitance [20], and a broad spectrum of antimicrobial activity against different micro-organisms [21].

Modification of the metallic surface—for instance, by the interposition of antibacterial agents, i.e., implant materials—is one of the methods of generating antibacterial properties [22]. Studies have been conducted regarding the usage of the element copper in steel, a process of producing material that can be used to fabricate antibacterial steels with very high antibacterial properties [23], as well as CuNPs coated onto metal alloys that exhibited antibacterial features [24]. In addition, NPs can be used as corrosion inhibitors [25], as well as nanoreservoirs for carrying inhibitors to the metallic surface [26].

As already mentioned above, the inhibition of amino acids for metal corrosion under various conditions is a matter that has aroused growing interest. Electrochemical methods such as potentiodynamic polarization, electrochemical impedance spectroscopy (EIS), and X-ray powder diffraction (XRD), as well as X-ray photoelectron spectroscopy (XPS), and scanning electron microscopy combined with an electron microprobe (SEM–EDS), are frequently used in corrosion studies [3,4,5,7,8,10,11]. However, Fourier-transform infrared (FT-IR) and Raman (RS) spectroscopies can also play an important role in the examination of various structural constituents and in the investigation of corrosion products formed on the surface of corroded materials [27,28]. Despite the wide range of studies of corrosion, there is still very little known about the structural changes to inhibitors deposited onto a metal surface, especially at the nanoscale. Progress in this area could provide some insight, allowing for a better understanding of corrosion and inhibition processes. Spectroscopic techniques such as surface-enhanced Raman spectroscopy (SERS) and surface-enhanced infrared absorption spectroscopy (SEIRA) tend to be regarded as a very promising tool to study the conformational changes of potential inhibitors on a metallic surface. Additionally, using a combination of atomic force microscopy (AFM) with infrared spectroscopy (IR) provides an opportunity to perform chemical characterization at nanometer spatial resolution. Previously published data has confirmed that AFM–IR applied for the investigation of the adsorption geometry of a molecule onto a metal surface ensures comparable results to the conventional SEIRA technique. However, investigation by means of AFM–IR together with the polarized light provided more precise details regarding molecule orientation against the metal surface [29,30,31].

With SERS, the effective Raman cross-section can be increased by many orders of magnitude (up to 10^6^–10^14^), meaning it is possible to obtain information even from a single molecule adsorbed onto the metallic surface [32]. Although the enhancement factor in SEIRA effect only increases the signal intensity by two/three orders of magnitude, the cross-section for infrared absorption is several-fold higher than the corresponding Raman cross-section [33]. SEIRA is explained as being the result of the enhanced optical field at the metallic surface, where the surface phonon resonance frequencies for the appropriate roughness of the used substrate are generated [33,34]. The phonon resonance effect on which SEIRA is based is similar to the plasmon resonance that produces the SERS signal. Generally, two mechanisms based on electromagnetic (EM) and chemical (CM) enhancement provide enhancement in SERS [33]. In the case of SEIRA, by way of analogy to SERS, the EM and CM mechanisms contribute to the total enhancement [35,36].

The combination of AFM and IR techniques offers advantages that are provided by the AFM and IR techniques separately [37,38]. The diffraction limit can be overcome using AFM–IR spectroscopy, where the infrared light generates a thermal expansion within the sample. As well as registering the infrared absorption intensities, the AFM tip detects the thermal expansion signal, which is directly proportional to the coefficient of sample absorption. The AFM–IR spectral patterns are correlated with those obtained by conventional IR spectroscopy [37,38,39,40].

The spectroscopic techniques discussed above are very selective, nondestructive, and enable studies of subtle changes in the orientation of the molecule through the process of adsorption onto the corroded metallic surface [29,30,31,41,42]. In addition, the effective range of signal enhancement is limited to the close proximity of molecular fragments to the metallic surfaces. The geometry and orientation of the molecules adsorbed onto metallic surfaces can be deduced based on these interactions. SERS, SEIRA, and nano-SEIRA are promising techniques applied in various fields including biomedicine, catalysis research, and biotechnology [43,44,45,46,47,48]. They can be also very useful in corrosion studies, especially in studies of the mechanism of the corrosion inhibition process, at the micro- and nanoscale [49,50,51].

In this paper, the use of spectroscopic methods such as FT-IR, RS, and techniques based on surface-enhanced effects such as SEIRA and SERS allowed for detailed examination of adsorption process of the amino acid, Thr, onto a corroded Fe surface with deposited CuNPs using the Inert Gas Condensation technique (IGC). In addition, the application of a novel nano-SEIRA technique which combines the chemical information provided by vibrational spectroscopy with the nanoscale spatial resolution and the topography of the investigated surface obtainable with AFM will allow detailed investigations of the molecule behavior at the metal surface. Moreover, it is possible to gain a better understanding of the interaction between molecule and metal at the nanolevel. 

## 2. Materials and Methods 

### 2.1. Preparation of Samples

Copper nanoparticles (CuNPs) were deposited by a cluster source (Mantis Deposition Ltd., Thame, UK) which combines magnetron sputtering with the Inert Gas Condensation technique. With this method, atoms of the target are ejected due to bombardment of Ar ions and then condensate into nitrogen-cooled aggregation zone. In that zone, the evaporated atoms collide with the gas atoms, lose kinetic energy, and form nanoparticles. Detailed information about the system can be found in the article by Kusior et al. [13]. The deposition process was carried out at a constant pressure of 5.7 × 10^−4^ Torr. The base pressure of the system was approximately 5.4 × 10^−8^ Torr. Nanoparticles were deposited from the copper target (MaTecK GmbH, Jülich, Germany) at a constant target current of 110 mA. The Cu source operated in direct current mode. The argon flow rate was constant during whole deposition process (100 sccm). Deposition time was 20 min. All these parameters were optimized prior to the final experiments and the selection of this configuration of deposition parameters was based on previous tests indicating good quality of the negatively charged copper nanoparticles produced [13,52]. Nanoparticles of about 4 nm in size were deposited onto Fe and Si substrates. The proper size of the nanoparticles was obtained thanks to mass filters which were placed in line with the cluster source. 

Prior to nanoparticle deposition, the Fe substrates (99.99%, 10 mm × 15 mm × 10 mm) were cleaned mechanically with the use of sandpaper sheets (SiC) of different grits (400–4000) and in an ultrasonic bath with ethanol (analytical grade) for 30 min each consequently. The substrates were then washed with deionized water and dried.

Thr was purchased from Sigma-Aldrich and used without further purification. Samples of Fe with deposited Cu nanoparticles (CuNPs-Fe) were immersed in 0.5-M HCl solution (reference sample) and 0.5-M HCl solution containing 10^−1^-M and 10^−3^-M Thr for 24 h. The HCl solutions were prepared from analytical grade reagent (AR) chemicals and deionized water (18 MΩcm^−1^).

### 2.2. AFM Measurements

AFM topographies were measured with a NT-MDT microscope (NT-MDT, Moscow, Russia) using commercially available silicon probes (135 μm nominal length, 30 μm nominal width) with a resonance frequency of 90 kHz and a force constant of 1.74 N/m. 

### 2.3. FT-IR and SEIRA Measurements

FT-IR and SEIRA spectra were recorded for a dried samples of CuNPs-Fe without and with Thr. The FT-IR spectrum were carried out for a dried solution of a 10^−1^-M Thr droplet on the calcium fluoride optical window. FT-IR and SEIRA spectra were recorded employing a Vacuum FT-IR VERTEX 70 V spectrometer (Bruker, Ettlingen, Germany) combined with the HYPERION 3000 IR microscope (Bruker Optics, Germany). The microscope was equipped with a liquid-nitrogen-cooled MCT (Mercury–Cadmium–Telluride) detector and a 15× magnification objective. The spectra were measured in reflectance mode covering the spectral range from 4000 cm^−1^ to 800 cm^−1^ (256 number of scans and 4 cm^−1^, spectral resolution). The spectra from the SEIRA were almost identical, except for small changes in the inten sities of some of the bands. In order to confirm the reproducibility of the observed phenomenon, the SEIRA spectra were recorded from three different areas. 

### 2.4. RS and SERS Measurements

RS and SERS spectra were collected for dried substrates (CuNPs-Fe samples) without and with Thr. The RS spectrum was performed for a dried solution of a 10^−1^-M Thr droplet on the glass. All spectra were recorded using InVia Renishaw Raman spectrometer (Renishaw, Wotton-under-Edge, United Kingdom) equipped with a CCD camera and confocal microscope. The excitation wavelength was provided by a He-Ne laser emitting at 632.8 nm with an 1800 grooves/mm grating. The power of the laser at the sample was set at approximately 15 mW. All presented spectra were collected with a spectral resolution of 1 cm^−1^ and in the 4000–100 cm^−1^ spectral range. The spectra were measured from 8 points of the sample (4 scans and 30 s of exposure time was enough to obtain good signal-to-noise ratio). The spectra from the SERS were almost identical, which indicates high reproducibility of the data obtained. While performing the measurements, no spectral changes that suggested desorption or decomposition of the sample were observed. 

### 2.5. Nano-SEIRA Measurements

Samples for the nano-SEIRA measurements were prepared in an identical manner to those for the transmission of SEIRA measurements. Nano-SEIRA spectra were recorded from three different areas using the NanoIR2 Anasys Instrument (Santa Barbara, CA, USA). For each of the investigated areas, fifteen spectra were considered. The signals obtained from the collected series were almost identical. An OPO (Optical Parametric Oscillator) laser tunable within the 1900 cm^−1^ to 900 cm^−1^ range (4 cm^−1^ spectral resolution, 256, an averaged number of pulses for each data point) was used as the infrared radiation source. When performing the measurements, 2% of the nominal incident laser power (8 W) was used. The spectra obtained were smoothed using a second-order, three-point Savitzky–Golay function. The AFM topographies were acquired in contact mode using a 13 kHz resonance frequency cantilever (0.07 N m^−1^ spring constant and 256 pixel resolution in the *x*/*y* directions). The surface condition of the sample was monitored during the measurements (the possibility of thermal damage within the samples was excluded).

### 2.6. Data Analysis

Interpretation and analysis of the recorded FT-IR, RS, SEIRA, and SERS spectra were performed using OMNIC (9.0 version) and OPUS (7.5 version). The interpretation of the nano-SEIRA results was done using Analysis Studio software (3.11.5715 version).

### 2.7. Fitting Procedure

Fitting of the 1650–1520 cm^−1^ spectral range for the SERS spectra and fitting of the 1680–1460 cm^−1^ spectral range for the SEIRA spectra of Thr (10^−1^ M) adsorbed onto the corroded CuNPs-Fe surface were performed using OMNIC software. A Gaussian-Lorentzian and Gaussian band shape were assumed and fixed for the SERS and SEIRA bands, respectively.

## 3. Results and Discussion 

**Figure 1:**
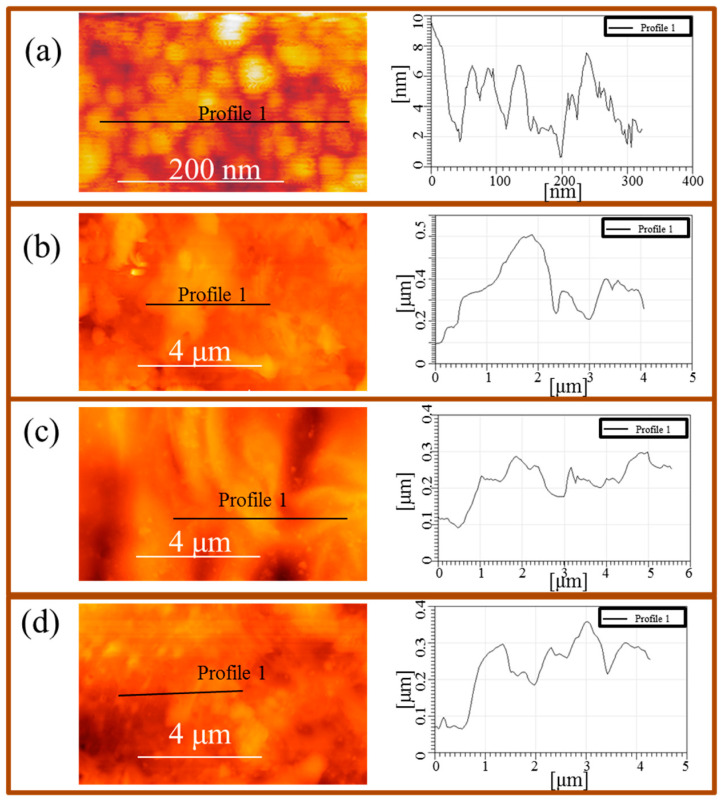
(**a**) Atomic force microscopy (AFM) images with height profiles of the copper nanoparticles (CuNPs)-Fe surface before corrosion; (**b**) corroded CuNPs-Fe surface (after 24 h of exposure to 0.5 M HCl); (**c**) corroded CuNPs-Fe surface with presence of Thr (10^−1^ M); (**d**) corroded CuNPs-Fe surface with presence of Thr (10^−3^ M).

The AFM technique was used to monitor topographical changes on the CuNPs-Fe surface after immersion in 0.5-M HCl solution without and with different concentrations of Thr. Figure 1 presents the AFM images of the reference sample (Fe surface with deposited CuNPs (CuNPs-Fe)) (Figure 1a), the corroded CuNPs-Fe surface (Figure 1b), and the corroded CuNPs-Fe surface in the presence of Thr (10^−1^ M) (Figure 1c) and Thr (10^−3^ M) (Figure 1d). As shown in Figure 1a, the spherical NPs of about 4–5 nm in size were deposited onto the Fe surface. Analysis of the size of the NPs was provided as a function of the height of the NPs due to the unavoidable broadening effect caused by the radius of the AFM tip [48] and, afterwards, the average NP size was calculated. Previous studies of CuNPs provided by Kusior et al. [13] indicated that application of the IGC technique allows for accurate determination of their size, morphology, chemical composition, and sputtering rate. The AFM image of the corroded CuNPs-Fe surface (Figure 1b) indicates the formation of a significant surface roughness (height of about several hundred nm) on the metallic surface due to the aggressive role of the chloride ions. The addition of Thr (Figure 1c,d) to the hydrochloric acid solution resulted in changes to the CuNPs-Fe surface, and surface smoothing was observed.

**Figure 2:**
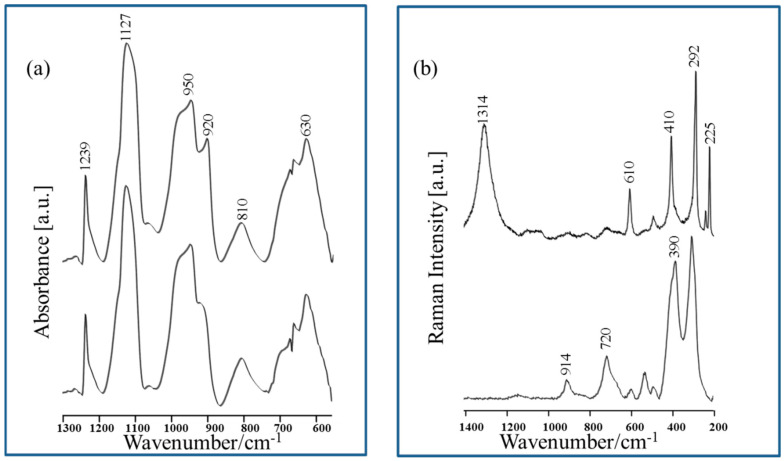
(**a**) The reference FT-IR spectra (top and bottom) of the corroded CuNPs-Fe sample. Measurement conditions: spectral range, 1300–550 cm^−1^; detector MCT (Mercury–Cadmium–Telluride). (**b**) The reference Raman spectroscopy (RS) spectra (top and bottom) of the corroded CuNPs-Fe sample. Measurement conditions: spectral range, 1400–200 cm^−1^; laser line, 632.8 nm.

The reference FT-IR and RS spectra obtained for the CuNPs-Fe surface after its immersion in 0.5 M HCl solution for 24 h are shown in Figure 2a,b, respectively. The FT-IR and RS spectra are dominated by bands which can be assigned to a corrosion product, which is a mixture of different compounds such as goethite (α-FeOOH), lepidocrocite (γ-FeOOH), feroxyhyte (δ-FeOOH), akaganeite (β-FeOOH), hematite (α-Fe_2_O_3_), and cuprite (Cu_2_O) [49,50,51,53,54,55,56]. In the FT-IR spectra (Figure 2a), the appearance of bands around at 920 cm^−1^, 810 cm^−1^, and 630 cm^−1^ can be assigned to α-FeOOH [49]. However, the presence of a significantly broadened band around 810 cm^−1^ and 630 cm^−1^ also suggests the formation of β-FeOOH on the corroded CuNPs-Fe surface [50]. In addition, the band with maximum at 1127 cm^−1^, with a shoulder at 1160 cm^−1^, and the broad band with a maximum at around 950 cm^−1^, mean the presence of lepidocrocite (γ-FeOOH) and feroxyhyte (δ-FeOOH) on the corroded CuNPs-Fe surface cannot be excluded [49,50,51,53]. Another overlapped band at 660 cm^−1^ in Figure 2a may suggest the formation of Cu_2_O on the corroded CuNPs-Fe surface [54,55].

The RS spectra recorded from the top part of the corroded CuNPs-Fe surface ((Figure 2b) presents the most characteristic and repeatable spectra) indicate the presence of α-Fe_2_O_3_ (bands at 410 cm^−1^, 292 cm^−1^, and 225 cm^−1^), γ-FeOOH (1314 cm^−1^ and 610 cm^−1^), and β-FeOOH (720 cm^−1^, 390 cm^−1^, and 312 cm^−1^) [49,51,52,53]. In addition, the appearance of bands at around 620 cm^−1^ and 530 cm^−1^ is characteristic for cuprite [54].

**Figure 3:**
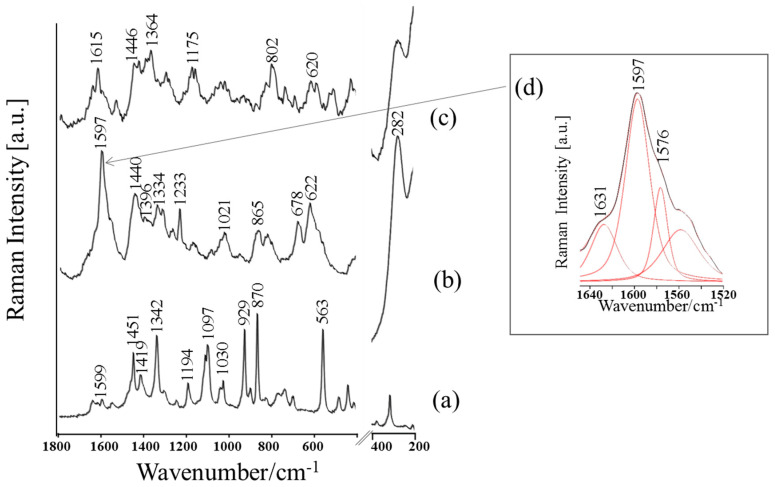
(**a**) RS spectrum of Thr (10^−^^1^ M/0.5 M HCl); (**b**) SERS spectra of Thr (10^−^^1^ M); and (**c**) Thr (10^−^^3^ M) adsorbed onto the corroded CuNPs-Fe surface. Measurement conditions: spectral range, 1800–200 cm^−^^1^; laser line, 632.8nm. (**d**) Inset: the deconvolution SERS spectrum; spectral range, 1650–1520 cm^−^^1^.

**Figure 4:**
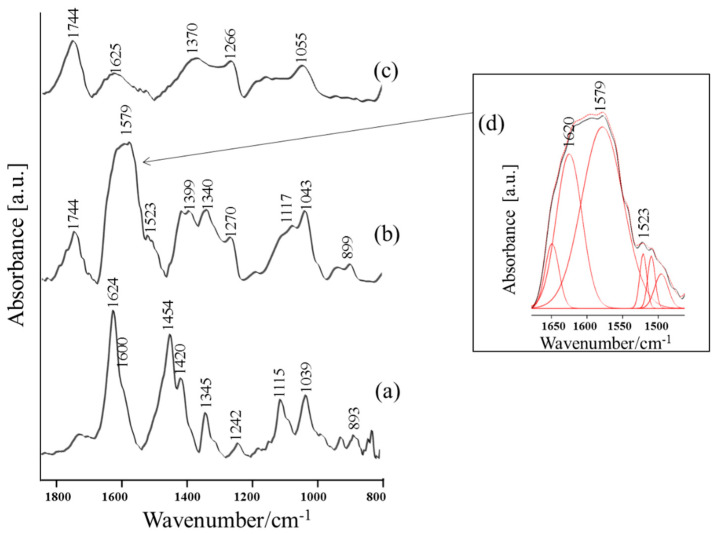
(**a**) FT-IR spectrum of Thr (10^−^^1^ M in 0.5 M HCl); (**b**) SEIRA spectra of Thr (10^−^^1^ M); (**c**) Thr (10^−^^3^ M) adsorbed onto the corroded CuNPs-Fe surface. Measurement conditions: spectral range, 1850–800 cm^−^^1^, MCT detector. (**d**) Inset: the deconvolution SEIRA spectrum; spectral range, 1680–1460 cm^−^^1^.

The reference RS and FT-IR spectra of the dried Thr (10^−1^ M Thr/0.5 M HCl) solution are shown in Figure 3a and Figure 4a, respectively. The proposed band assignments were based on extensive experimental and theoretical data from the literature on the molecular structure of Thr in different states under various conditions [57,58,59,60,61,62,63,64]. It is well known that the preparation of samples for the measurements may have an impact on the spectral information obtained. Many parameters determine the relative intensity of the specified bands in the vibrational spectrum, i.e., the state of the sample condition [65], solvent effect [66], and pH of the solution [15,66,67]. For this reason, the same sample preparation procedure was applied in all spectroscopic experiments. Table 1 summarizes the wavenumber and band assignments with full width at half maximum (FWHM) of selected vibrational modes of the studied system.

The RS spectrum of the dried Thr (10^−1^ M/0.5 M HCl) solution (Figure 3a) is dominated by characteristic modes of terminal groups which appear at 1641 cm^−^^1^ [δ_as_(NH_3_^+^)], 1599 cm^−^^1^ [ν_as_(COO)], 1547 cm^−^^1^ [δ_s_(NH_3_^+^)], 1419 cm^−^^1^ [ν_s_(COO)], 1246 cm^−^^1^ [δ(NH_3_^+^)], and 563 cm^−^^1^ [ρ_r_(COO)] (see Table 1 for detailed band assignments). In addition, C–H mode vibrations (1451 cm^−^^1^ [δ(CH_3_)], 1342 cm^−^^1^ [δ(CH_3_)/δ(CH)], 1302 cm^−^^1^ [δ(CH)], 1194 cm^−^^1^ [ρ_r_(CH)], CC (929 cm^−^^1^), and C–N skeletal stretching (870 cm^−^^1^, 902 cm^−^^1^) and C–O groups (1114 cm^−^^1^, 1030 cm^−^^1^) are observed.

Analogously, in the FT-IR spectrum of Thr (Figure 4b), the most characteristic bands are attributed to the terminal groups (1723 cm^−^^1^ [(ν(C=O)], 1624 cm^−1^ [δ_as_(NH_3_^+^)/ν_as_(COO)], 1420 cm^−^^1^ [(ν_s_(COO)]). The CH group and skeletal stretching vibrations can be also assigned to the 1454 cm^−^^1^, 1345 cm^−^^1^, 929 cm^−^^1^, and 893 cm^−^^1^ bands, respectively (see Table 1 for appropriate band assignments).

Figure 3b,c and Figure 4b,c present the SERS and SEIRA spectra obtained for dried CuNPs-Fe samples after 24 h immersion in 0.5 M HCl solution with a concentration of Thr set to 10^−^^1^ M and 10^−^^3^ M. The proposed band assignments and the FWHM values of the vibrational bands are presented in Table 1. As shown in the abovementioned figures, there are visible differences in the SERS and SEIRA spectral features such as relative intensity, width, and position of wavenumber, in comparison to those observed in the conventional RS and FT-IR spectra of Thr, respectively. The addition of Thr (10^−^^1^ M and 10^−^^3^ M) to the corrosive solution completely changed the spectral response. It was observed that amino acid adsorbed onto the metal surface. The SERS and SEIRA spectra are rich in bands which can be assigned to Thr (see Table 1 for appropriate bands assignments), which indicates that Thr interacts strongly with the CuNPs-Fe surface in a corrosive environment. 

The application of complementary techniques based on surface-enhanced effects, SERS and SEIRA, made it possible to obtain complete spectral information about the adsorption geometry of the bioinhibitor on an Fe surface with deposited negatively charged metallic nanoparticles [52] in the presence of aggressive environment.

Based on surface selection rules [68,69,70,71,72], it is possible to determine interactions between Thr and the CuNPs-Fe surface under certain acidic conditions. During the adsorbate interaction with metallic surface, bands which are clearly visible in the RS/FT-IR spectra can be invisible in the SERS/ SEIRA spectra. On the other hand, significantly enhanced bands can be observed in the SERS/SEIRA spectra which are very weak in the RS/FT-IR spectra.

The SERS spectrum of the investigated bioinhibitor (Thr with a concentration of 10^−^^1^ M) on the CuNPs-Fe surface (Figure 3b) has a strong and broad band with a maximum at 1597 cm^−^^1^ (the comparable relative intensity among the spectrum), which can be assigned to the asymmetric deformation modes of the amine [δ_as_ (NH_3_^+^)] and asymmetric stretching of carboxyl [ν_as_(COO)] groups. This spectral feature exhibits significant enhancement, red-shift (Δῡ = 2 cm^−^^1^), and substantial broadening (Δ_FWHM_ = 12 cm^−^^1^) in comparison to those observed in the normal RS spectrum of the nonadsorbed molecule. The presence of overlapping [δ_as_(NH_3_^+^)]/[ν_as_(COO)] bands in the same spectral region makes an unambiguous assignment of the abovementioned band difficult. It should be emphasized that, usually, amine groups are not strongly enhanced in RS spectra in the 1800–500 cm^−^^1^ region [73]. However, the appearance of shoulder at 1545 cm^−^^1^ due to symmetric deformation stretching mode of amine group (band very weak in the RS spectrum (Figure 3a) suggested that the protonated nitrogen in amine group interacts strongly with the CuNPs-Fe surface and blocked the formation of corrosion products. Furthermore, this finding is supported by the significant enhancement of the overlapped bands in the spectral region from 1631 cm^−^^1^ to 1360 cm^−^^1^. Guzzetti et al. [63] assigned these spectral regions in the calculated and experimental RS spectrum of Thr in aqueous solution mainly to the asymmetric and symmetric deformation modes of NH_3_. In the SERS spectrum (Figure 3b), the stronger enhancement of the broad band at 1440 cm^−^^1^ (Δῡ = 11 cm^−^^1^, Δ_FWHM_ = 16 cm^−^^1^) in comparison to the RS spectrum (Figure 3b) has a rather larger component of deformation vibration of the protonated amine group than methyl groups. In addition, the appearance of the band at 1233 cm^−^^1^ (very weak in RS spectrum, Figure 3a) can be assigned mainly to the deformation vibration of NH_3_^+^ [58]. All of the abovementioned bands are significantly enhanced, broadened, red-shifted, and exhibit higher relative intensities in relation to the free-molecule spectra.

In addition, the interaction of carboxylate group with the metallic surface cannot be excluded, although overlapping with other bands in this spectral region, the symmetric stretching COO band observed at 1399 cm^−^^1^ (see Figure 3b) is only slightly more enhanced in comparison to RS spectrum of nonadsorbed Thr. Talley et al. [67] observed that the relative intensity of the [ν_s_(COO)] band is dependent on the pH in the environment surrounding the nanoparticles and, together with lowering of pH, the number of dissociate carboxylate groups decreases; for this reason, the relative intensity of the [ν_s_(COO)] band can be reduced. In addition, based on the vibrational analysis provided by Guzzetti et al. [63], the enhanced bands at 865 cm^−^^1^ and 678 cm^−^^1^ are due to in-plane and out-of-plane COO deformation modes, respectively. Based on surface selection rules, it can be suggested that the carboxylate group is almost parallel to the metal surface (adsorbed through π-electrons) and assisted in the process of adsorption with metallic surface [70,71]. As a result, it is possible to say that the interaction between amine group and CuNPs-Fe surface brings the COOH or COO groups closer to the CuNPs-Fe surface.

On the other hand, the decrease in the intensity of spectral features of the group CH (1334 cm^−^^1^ [(δ(CH_3_)/δ(CH)], 1315 cm^−^^1^ [δ(CH)], 1168 cm^−^^1^ [ρ_r_(CH)]) upon adsorption suggests that these fragments are at some distance from the CuNPs-Fe sample.

Analysis of the SERS spectrum indicates that, at a lower amino acid concentration (10^−3^ M), the same set of bands appear (Figure 3c). Although, together with the decreasing concentration of amino acid from 10^−^^1^ M to 10^−^^3^ M, there is a decrease in the relative intensities of bands characteristic of the amine and carboxyl groups. The application of the inhibitor at a higher concentration results in a stronger interaction between Thr and the metal substrates. It has been reported that inhibition efficiencies increase as amino acid concentration increases [10,74]. Upon a decrease in concentration of Thr, there is a decrease in the relative intensity of the broad band with maximum at 1615 cm^−^^1^ due to [δ_as_(NH_3_^+^)]/[ν_as_(COO)] groups. In addition, SERS bands with maxima around 1364 cm^−^^1^ and 802 cm^−^^1^ can be attributed to the COO group. The appearance of the deprotonated carboxylic group is the result of interaction with the metallic surface, despite the low pH of intensity of the δ(COO) band at 802 cm^−^^1^ (see Figure 3c) in comparison to the band at 1364 cm^−^^1^ ([ν_s_(COO)]), this suggests that the terminal group of the Thr (10^−^^3^ M) adopts more/less-tilted orientation upon adsorption onto the CuNPs-Fe sample [71]. The abovementioned changes may be caused by weakening of the interaction between amine group and the metallic surface (decrease of relative intensity of the bands with a maximum at 1615 cm^−^^1^ and 1446 cm^−^^1^). 

In addition, the appearance of the band at 622 cm^−^^1^ in the SERS spectrum (Figure 3b) can be attributed to Cu(I)–O vibrations in Cu_2_O (however, the contribution to this band of wagging carboxylate vibration cannot be excluded). The presence of another band at 282 cm^−^^1^, which may correspond to a stretching Cu-O vibrational mode, indicates the formation of cuprite on the copper surface, which protects the surface against corrosion [55]. At the same time, the appearance of this strong band (stronger in SERS spectrum obtained at a higher amino acid concentration (10^−^^1^ M)) can be attributed to the existing strong metal–molecule interaction [70].

SEIRA results confirm the abovementioned considerations (Figure 4b). In the 1750–1370 cm^−^^1^ spectral region, which is significantly enhanced, the signal arises mainly from modes of terminal groups (see Table 1 for detailed bands assignment). In a way that is analogous to the SERS spectrum (Figure 3b), in the SEIRA spectrum (Figure 4b), both [δ_as_(NH_3_^+^)] and [ν_as_(COO)] modes overlapped and shifted significantly in comparison to FT-IR spectrum recorded for free Thr. It is worth noting that the broadening and red-shift in wavenumber of the most enhanced band with a maximum at 1597 cm^−^^1^—which show a few components (see inset (d) in Figure 4) in comparison to the FT-IR spectrum of free Thr (Figure 4a)—confirms the existing strong interaction between the abovementioned terminal groups and the metal surface. The FWHM values of the SEIRA bands due to terminal groups increased upon adsorption (see Table 1). The appearance of a shoulder on the abovementioned band at 1523 cm^−1^ due to the symmetric deformation stretching mode of the amine group indicates the existence of strong electrostatic adsorption of amine group onto negatively charged metallic surfaces [75]. It should be emphasized that in the literature [75,76], the absorption band corresponding to the δ_s_(NH_3_^+^) is often weakly enhanced or disappears after adsorption for many amino acids. However, Begonja et al. [77] observed this band and proposed that the amino group plays a significant role in the n process adsorption of cysteine onto the metallic surface. In addition, Ustunol et al. [15] observed that the protonated surface species of amino acids adsorbed onto metallic surface under acidic conditions. The significant enhancement of band at 1744 cm^−^^1^ (Δῡ = 21 cm^−^^1^, FWHM = 45 cm^−^^1^) due to –C=O stretching vibrations in comparison to FT-IR spectrum of Thr (Figure 4a) indicated the presence of carbonyl group in close proximity to the CuNPs-Fe surface. 

The presence of the –C=O band is typical of the undissociated carboxylate group and indicated that the protonation of the carboxylate group takes place at a highly acid pH [64]. The stronger enhancement of band at 1043 cm^−^^1^ [ν(C–OH)] in comparison to FT-IR spectrum of free molecule (Figure 4a) confirms this observation. 

Upon a decrease in Thr concentration (10^−3^ M), in a way analogous to SEIRA spectra under the same conditions, the weakening of the interaction between the terminal group and the metallic surface (decrease of relative intensity of overlapped bands with maximum about 1620 cm^−^^1^) is observed.

**Figure 5:**
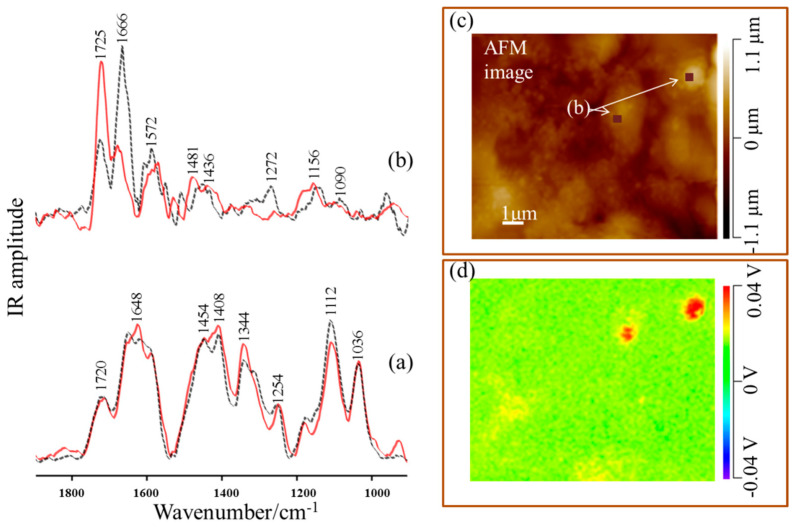
(**a**) Nano-IR spectra of the dried sample of Thr (10^−^^1^ M/0.5 M HCl); and (**b**) nano-SEIRA spectra of the corroded CuNPs-Fe surface with presence of Thr (10^−^^1^ M) recorded for *p-*(black color and dotted line) and *s*-polarization modulation (red color and solid line) in the spectral range of 1900–900 cm^−^^1^; (**c**) AFM image of Thr 10^−^^1^ M adsorbed onto the CuNPs-Fe surface; (**d**) the intensity map of the 1730–1580 cm^−^^1^ spectral range of the CuNPS-Fe–Thr complex.

The application of nano-SEIRA technique made it possible to observe subtle reorientation of molecule upon adsorption onto the CuNPs-Fe surface. Figure 5 presents the nano-SEIRA spectra of Thr adsorbed onto metal surface recorded for *s*- and *p*-polarization modulations including the AFM image. The wavenumber and proposed band assignments for the representative nano-SEIRA spectra are shown in Table 1. 

The nano-SEIRA spectra collected for two orthogonal polarizations were measured from the same surface area. In addition, no normalization for the investigated spectral regions was used. The spectra presented in Figure 5b correspond to an averaged signal recorded from the selected area of the investigated sample. The use of NanoIR2 configuration with the OPO laser which is located at 15˚ to the surface provided the opportunity to establish the bonds located almost parallel to the investigated metal substrate and perpendicular to the investigated surface. With *s*-polarization modulation, which is normal to the tip axis, the vibrational features responsible for parallel or tilted band positions will be significantly enhanced. In contrast, *p*-polarized light (parallel to the tip axis) provided a strengthening of the spectral signals with dipoles adopting a position perpendicular to the investigated metal surface [25,26,27,33,36].

The reference spectrum of Thr (Figure 5a) for the dried sample at a concentration of 10^−^^1^ M Thr/0.5 M HCl, collected using the NanoIR2 configuration with the OPO laser in two orthogonal polarization modulations, provided a good match with FT-IR spectrum of amino acid (Figure 4a). Additionally, very good compatibility between the nanoscale *p*-polarized SEIRA (Figure 5b, black color and dotted line) and the conventional SEIRA spectrum (Figure 4b) was observed.

**Figure 6:**
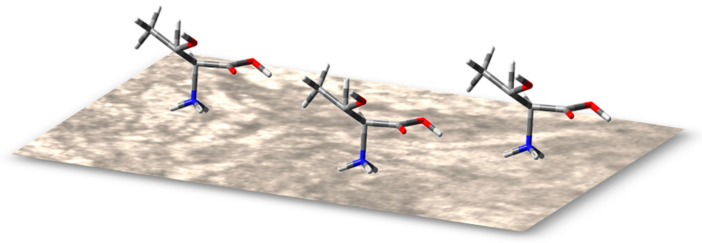
The schematic orientation of Thr (10^−^^1^ M) onto the CuNPs-Fe surface in a corrosive environment.

Both the *s*- and *p*-polarized nano-SEIRA spectra contain the same set of bands as the SEIRA spectrum, with bands at 1725 cm^−^^1^, 1666 cm^−^^1^, 1572 cm^−^^1^, 1481 cm^−^^1^, 1436 cm^−^^1^, and 1272 cm^−^^1^, which are attributed to the terminal groups of Thr (Table 1). This observation confirms a strong interaction between terminal groups and the Cu nanoparticles deposited onto the Fe surface. Figure 5c presents the AFM topography and nano-IR image of the 1725 cm^−^^1^ band distribution. The significant enhancement of the 1725 cm^−^^1^ spectral feature due to the carbonyl C=O group in the *s*-polarized spectrum suggests an almost parallel orientation of this bond, and, together with the appearance of the band at 1572 cm^−^^1^ and 1436 cm^−^^1^, indicates a more or less flat orientation of the carboxyl group from Thr (10^−^^1^ M) onto CuNPs-Fe surface, which reaffirms the assumptions based on SERS. In addition, the stronger amplification of band at 1666 cm^−^^1^ in the *p*-polarized nano-SEIRA spectrum suggest that the amine group strongly interacts with metal surface and adopts a perpendicular orientation on the metal surface. The studies presented here show that Thr bind to the corroded CuNPs-Fe surface by the terminal group of amino acid. The suggested orientation of Thr (10^−1^ M) onto the CuNPs-Fe surface is presented in Figure 6.

## 4. Conclusions

In this study, vibrational spectroscopy techniques were applied to perform a complex investigation of an Fe surface modified by deposited Cu nanoparticles of about 4–5 nm in size in the presence and absence of amino acid. The addition of threonine to the hydrochloric solution had a strong influence on the metal surface, and AFM images of the corroded CuNPs-Fe surface with the presence of 10^−^^1^ M Thr indicated a significant surface smoothing of the outer layer. The application of complementary surface-enhanced vibrational techniques such as SERS, SEIRA, and a novel approach of combining IR and AFM as well as nano-SEIRA techniques, made it possible to provide a precise description of adsorbate binding to the metal surface in an aggressive environment. The SERS, SEIRA, and nano-SEIRA data allowed us to conclude that the protonated amine and carboxylate groups have a strong influence on the binding mechanism of Thr at the CuNPs-Fe surface. The studies presented confirmed that there is a very good match between the spectra recorded by SERS, SEIRA, and nano-SEIRA techniques.

The comprehensive spectroscopic analysis of the process of adsorption of the potential bioinhibitor onto an Fe surface modified by Cu nanoparticles proved to be very important for investigating the molecular behavior at the metal surface. In addition, this approach allowed for a better understanding of the amino acid/metal interaction at the nanolevel in the corrosive environment. The findings suggest that the application of novel spectroscopic methods has great potential for the complex investigation of phenomena related to the adsorption of inhibitors onto new materials. 

## Figures and Tables

**Table 1:** Wavenumber and proposed band assignments for Raman spectroscopy (RS), surface-enhanced Raman spectroscopy (SERS), Fourier-transform infrared (FT-IR), surface-enhanced infrared absorption spectroscopy (SEIRA), and nano-SEIRA spectra of Thr (10^−1^ M) adsorbed onto the corroded CuNPs-Fe sample.

Band Assignments	RS		SERS		FT-IR		SEIRA		Nano-SEIRA	
	ν (cm^−^^1^)	FWHM (cm^−1^)	ν (cm^−1^)	FWHM (cm^−1^)	ν (cm^−1^)	FWHM (cm^−1^)	ν (cm−1)	FWHM (cm^−1^)	*s*-pol	*p*-pol
ν(C=O)	−	−	−	−	1723 vw	30	1744 m	45	1725 vs	1724 m
δ_as_(NH_3_^+^)	1641 vw	15	1631 sh	23	1624 vs	39	1620 sh	54	1666 vs	1672 m
δ_as_(NH_3_^+^)/ ν_as_(COO)	1599 vw	16	1597 vs/ 1576 sh	28 20	1598 sh	27	1579 vs	68	1572 m	1572 m
δ_s_(NH_3_^+^)	1547 vw	13	1545 sh	26	−	−	1523 sh	23	−	−
δ(CH_3_)/ δ(NH_3_^+^)	1451 m	9	1440 s	25	1454 vs/ 1420 sh	40 44	1450 sh	60	1481 m	1468 m
ν_s_ (COO)/ δ(CH)	1419 m	16	1396 sh	27	1385 sh	22	1399 s	28	1436 sh	1438 sh
δ(CH_3_)/ δ(CH)	1342 s	15	1334 m	7	1345 m	46	1340 m	63	−	−
δ(CH)	1302 sh	27	1315 m	20	1320 sh	29	−	−	−	−
δ(NH_3_^+^)/ ν(C-OH)	1246 vw	11	1233 m	13	1242 w	49	1270 sh	34	1272 m	1260 w
ρ_r_(CH),ν(C-C), δ(OH)	1194 m	11	1168 w	8	1180 vw	47	1200 w	36	1156 m	1140 w
δ(C-O) ν(CC), δ(NH)	1114 sh	22	−	−	1115 m	43	1117 sh	45	−	−
ν(C-O)	1097 s	16	−	−	1086 sh	19	−	−	1090 w	1089 w
ν(C-OH), ν(C-N)	1030 m	9	1021 m	27	1039 m	26	1043 m	35	−	−
ν (CC)	929 s	9	−	−	929 w	53	945 w	34	−	−
ν(C-N), ν(C-C)	902 w	11	−	−	893 w	35	899 w	28	−	−
ν(CCN)/ δ_ip_(COO)	870 s	8	865 m	20	−	−	−	−	−	−
δ(COO)	−	−	823 m	18	−	−	−	−	−	−
δ_oop_(COO)			678 m	35						
ν(Cu-O), ρ_w_(COO)	−	−	622 m	23	−	−	−	−	−	−
ρ_r_(COO)	563 s	12	−	−	−	−	−	−	−	−
ν(Cu-O)/ Cu-Thr	−	−	282 s	45	−	−	−	−	−	−

Abbreviations: ν—stretching; δ—deformation; s/as—symmetric and asymmetric; ρr—rocking; ρw—wagging; ip—in plane vibrations; oop—out of plane vibrations; Thr—threonine.
